# Stromal protein CCN family contributes to the poor prognosis in lower-grade gioma by modulating immunity, matrix, stemness, and metabolism

**DOI:** 10.3389/fmolb.2022.1027236

**Published:** 2022-12-16

**Authors:** Zhihui Liu, Jiasheng Wu, Hang Ji, Hongtao Zhao, Fang Wang, Jiawei Dong, Jiheng Zhang, Nan Wang, Xiuwei Yan, Kaikai Wang, Shaoshan Hu

**Affiliations:** ^1^ Cancer Center, Department of Neurosurgery, Zhejiang Provincial People’s Hospital, Affiliated People’s Hospital, Hangzhou Medical College, Hangzhou, Zhejiang, China; ^2^ Department of Neurosurgery, West China Hospital, Sichuan University, Chengdu, Sichuan, China; ^3^ Department of Neurosurgery, The Second Affiliated Hospital, School of Medicine, Zhejiang University, Zhejiang, Hangzhou, China; ^4^ Key Laboratory of Precise Treatment and Clinical Translational Research of Neurological Diseases, Hangzhou, Zhejiang, China

**Keywords:** extracellular matrix, metabolism, stemness, immunity, lower-grade glioma

## Abstract

**Background:** The CCN family of stromal proteins is involved in the regulation of many important biological functions. However, the role of dysregulated CCN proteins in lower-grade glioma (LGG) remain less understand.

**Methods:** The clinical significance of the CCN proteins was explored based on RNA-seq profiles from multiple cohorts. A CCNScore was constructed using LASSO regression analysis. The PanCanAtlas data and MEXPRESS database were employed to elucidate molecular underpinnings.

**Results:** The expression of CCN4 was associated with poor prognosis in LGG. The CCNScore (CCN1 = 0.06, CCN4 = 0.86) showed implication in prognosis prediction, subtype assessment and therapy selection. The gene mutation pattern of the high-CCNScore group was similar with glioblastoma, including EGFR, PTEN, and NF1 mutation frequently. Besides, the high-CCNScore group was comprised of samples mainly classic-like and mesenchymal-like, had lower methylation levels, higher stemness, higher inflammation, higher levels of extracellular matrix remodel and dysfunction of metabolic pathways. On the other hand, the low-CCNScore group consisted mainly of IDH-mutation LGG, and was characterized by TP53, CIC, and ATRX gene mutations, hyper-methylation status, lower stemness, lower proliferation, immune quietness and low extracellular matrix stiffness.

**Conclusion:** In summary, these results outlined the role of CCN family in LGG and provided a potential and promising therapeutic target.

## Introduction

Lower-grade glioma (LGG) accounts for 43.2% of central nervous system (CNS) glioma. Despite standard treatment, most patients experience tumor recurrence or progression (Weller et al., 2021). Recently, immunotherapy is revolutionizing cancer treatment, but seem meet its Waterloo in the treatment of glioma (Sampson et al., 2020). The reason for this phenomenon may be related to the unique tumor microenvironment (TME) of glioma, including the specific resident cell type, the extracellular matrix (ECM) composition and metabolic distinctions (Quail and Joyce, 2017). Therefore, identification of therapeutic targets from the unique glioma TME was promising. Distinct from other collagen- and laminin-rich tissues, the structural ECM of the brain is uniquely composed of glycoproteins, proteoglycans, and glycosaminoglycans (Winkler et al., 2020). Stiff ECM lead to increased hypoxia and promotes aggressiveness of glioma, which in turn aggravate the ECM stiffness and was associated with the radiotherapy and immunotherapy tolerance (Chaudhuri et al., 2020). Therefore, detailed characterizing the structural ECM remodeling and potential molecular mechanism are warrant.

Stromal cell proteins (SCPs), which are non-structural ECM, can remodel the tissue by binding to structural ECM. SCPs are crucial regulators of ECM homeostasis and are frequently found to be dysregulated in various malignancies (Thakur and Mishra, 2016). Unlike the tumor cells themselves, these SCPs hardly mutate as the tumor progresses (Valkenburg et al., 2018). Therefore, targeted therapy against SCPs is attractive. The CCN family, an important component of the secretory ECM-related protein family, consists of six family members, Cyr61/CCN1 (cysteine-rich 61), CTGF/CCN2 (connective tissue growth factor), Nov/CCN3 (nephroblastoma overexpressed), and Wnt pathway proteins (WISP1/CCN4, WISP2/CCN5, and WISP3/CCN6) (Jun and Lau, 2011). The family members share 40%–90% homologous sequences, and the common structure contains an N-terminal secretory signal peptide and four functional domains. Based on their multifunctional four-module structure, the CCN family is involved in vital activities such as cell proliferation, differentiation, chemotaxis, adhesion, angiogenesis, and ECM formation, and is inextricably associated with tumor progression (Perbal, 2004). Additionly, a recent research has revealed dual roles for WISP1 in maintaining glioma stem cells (GSCs) as well as tumor-associated macrophages (Tao et al., 2020). However, the critical roles of CCN proteins with broad functions in LGG and more possible mechanisms of CCN proteins in cancer needs further investigation.

To fill this gap, the clinical implication of the CCN family was explored, including the relationship with patient prognosis, pathological classification, histological subtype, molecular subtype, and clinical response. Next, the CCNScore was constructed to create a clinically feasible riskscore system in predicting the prognosis for patients with LGG and to guide the selection of treatment. Then, the relationship between CCNScore with the genomic and epigenomic features in LGG and with tumor malignant phenotypes including stemness, genetic instability and metabolic disorders etc was explored. Moreover, the effects of CCNSore on TME, including on stromal cells, ECM, immune cells, immune molecules, immune antigen and its presentation and immune escape was elucidated. Finally, multi-omics assay was employed to overview the regulation of the CCN family in LGG, including gene mutations, copy number variations (CNV), DNA methylations, transcript number, promoter information and protein interactions. Our study highlighted the role of CCNs in LGG and provided a promising therapeutic target for LGG.

## Materials and methods

### Preparation of data

RNA-seq expression profiles and corresponding demographics were retrieved from TCGA (The Cancer Genome Atlas, https://portal.gdc.cancer.gov/, and CGGA Chinese Glioma Genome Atlas, http://www.cgga.org.cn/database. RNA-seq data of normal cerebral cortex were obtained from the GTEx database (https://www.gtexportal.org). Data were screened for origin in Brain - Cortex and Brain - Frontal Cortex (BA9). Clinical profiles and RNA-seq data of pan-cancer were obtained from the UCSC Xena database (http://xena.ucsc.edu/). The remaining clinical profiles, molecular subtypes, genomic signatures and multi-omics data of LGG and pan-caner was obtained from PanCanAtlas Publications (https://gdc.cancer.gov/about-data/publications/pancanatlas). Cancer-associated promoter genes were downloaded from the Cistrome Cancer database (http://cistrome.org/CistromeCancer/). In addition, batch effects of data from different databases were removed using “normalizeBetweenArrays” function of the R package “limma”.

For the CCN family, single nucleotide variants (SNV) data were obtained from the TCGA database, CNV data were obtained from the UCSC Xena database, and methylation profile, transcript number and clinical traits were obtained from the MEXPRESS database (https://mexpress.be/index.html). The protein-protein interaction (PPI) network was generated from the STRING database (https://www.string-db.org).

### Multi-omics analysis

The expression of CCN family genes was compared between 207 normal cerebral cortex tissues and 529 TCGA-LGG samples and 590 CGGA-LGG samples using the R package “limma”. Differences in gene mutation types and frequencies were summarized and exhibited using the R package “maftools”. Gene location, amplification and deletion, and corresponding frequencies plots were exhibited in the circle plot. Cor-value >0.2 and *p*-value <0.001 were set as the cutoff of screen candidate cancer promoter genes and the Sankey diagram was drawn using the “ggalluvial” package.

### Construction of CCNScore

We constructed a riskscore model based on the CCN family with the “LASSO” regression method. The 10-fold cross-validation was used to verify the accuracy of the model. After 1000 iterations, we calculated the riskscore, also known as CCNScore, quantify the prognostic impact of differentially expressed CCN family genes. CCNScore was calculated by the following equation: $CCNScore=\begin{array}{c} n\\ i=1 \end{array}Coef\left({CCNs}_{i}\right)*\exp\left({CCNs}_{i}\right)$ , where Coef refers regression coefficient (lambda min) and exp indicates gene expression. When constructing the CCNScore, 70% of the TCGA samples were randomly selected as the train set and the remaining 30% as the test set. In addition, data from CGGA were not involved in the construction of the CCNScore and were therefore used as external validation set.

### Survival and clinical traits correlation analysis

The optimal cutoff values of CCNScore were calculated using the “surv_cutpoint” function of the “survminer” package for sample stratification. Overall survival (OS), progression-free interval (PFI), disease-specific survival (DSS) and disease-free interval (DFI) were used as outcome indicators, respectively. The R packages “survial” and “survminer” were employed for survival analysis. In addition, univariate and multivariate cox regression analysis were employed to determine the independent predictive significance. The “timeROC” package was used to plot ROC curves for their prognostic accuracy analysis, and the “rms” package was used to construct a nomogram.

### Functional enrichment and single-sample gene-set enrichment analysis

The “clusterProfiler” package was used to perform enrichment analysis based on gene ontology (GO) and KEGG terms. Terms with FDR q values <0.05 were considered significant. The single sample gene set enrichment analysis (ssGSEA) analysis was performed using the R package “GSVA”, and 29 immune signatures were used to detect immune phenotypes, including cell types, functions and pathways (Charoentong et al., 2017). To discover the underlying mechanisms in different subgroups, typical biological processes were quantified by ssGSEA. We introduced ten oncogenic pathway gene sets, tumor microenvironment core pathway gene sets, metabolic pathway gene sets, DDR gene sets, extracellular matrix structural component sets, immunogenic death, and EMT gene sets into our analysis, as shown in Supplementary Table S1.

### Acquisition of epigenetic data and stemness indices

Pan-cancer methylation subtype (Hoadley et al., 2018) and pan-glioma methylation subtype (Ceccarelli et al., 2016) were obtained from previous studies. Glioma stem cell markers were obtained from published single cell-seq files (Suva and Tirosh, 2020). The TCGA team used the “OCLR” algorithm to quantify the stemness of the tumor samples and obtained two stemness indices, in which the mRNA expression-based stemness index (mRNAsi) reflects the gene expression characteristics of stem cells and the methylated DNA-based stemness index (mDNAsi) reflects the epigenetic characteristics of stem cells. Epigenetic regulation-based mRNAsi and mDNAsi (EREG-mRNAsi and EREG-mDNAsi) were obtained by reconstructing the gene regulatory network from methylation and transcriptome data using the “ELMER” package and using the identified features as input to the “OCLR” (Malta et al., 2018).

### DNA damage signature

DNA damage-related signatures were collected from Genomic Data Commons-panimmune, as shown in Supplementary Table S1. In this study, tumor purity, leukocyte fraction (LF), cancer cell ploidy, loss of heterozygosity (LOH), and CNV burden were introduced to quantify DNA damage. The homologous recombination defect (HRD) score contained a composite score and a component score (Knijnenburg et al., 2018). Copy number variants and mutations were integrated to quantify intra-tumor heterogeneity (ITH) according to clonality by the “ABSOLUTE” algorithm. The aneuploidy score was formulated as the sum of the deleted or amplified chromosome arms (Taylor et al., 2018). The enrichment level of DNA damage-related signatures in each sample was finally evaluated by the ssGSEA analysis.

### Analysis of tumor microenvironment

Information on pan-cancer immunophenotype, immune molecules including stimulatory factors, inhibitory factors, HLA, immune checkpoint and antigenic peptide load were obtained from previous study (Thorsson et al., 2018). ESTIMATE is a new gene expression signature-based algorithm that analyzes the infiltration fraction of immune and stromal cells based on the RNA-seq expression profile. In addition, the “CIBERSORT” algorithm deconvolve the infiltration of 22 immune cells in the tumor microenvironment. We used the “xcell” package to calculate the content of stromal cells in tumor samples. The TISCH (http://tisch.comp-genomics.org/home/) database was employed to analyze the origin of CCN4 at single-cell resolution.

### Immune escape

The association between the CCNScore and LGG immune damage and invasion was analyzed through the Tumor Immune Dysfunction and Exclusion (TIDE) (http://tide.dfci.harvard.edu/). TIDE predicts the efficacy of immune checkpoint inhibitors through a comprehensive analysis of several tumor expression profiles and can determine the presence of cancer-associated fibroblast (CAF) and myeloid-derived suppressor cell (MDSC) inhibiting T cell infiltration in immune-cold tumors.

### Statistics

All statistics were performed using R software (version 4.0.2). Wilcoxon test and Kruskal-Wallistest were employed to compare continuous variables between groups. Chi-square test and fisher’s exact test were used to compare composition ratios. Pearson correlation test was performed for co-expression analysis and correlation analysis. Survival curves were plotted using the Kaplan-Meier (K-M) analysis and log-rank test was used to detect survival differences. Among the results calculated by all statistical methods above, *p* < 0.05 was considered statistically significant. Fisher’s exact test was used to calculate and identify driver genes for the High- and Low-CCNScore subgroups, and *p* < 0.05 was considered significant.

## Results

### Validation of the value of CCN proteins in lower-grade glioma

**FIGURE 1:**
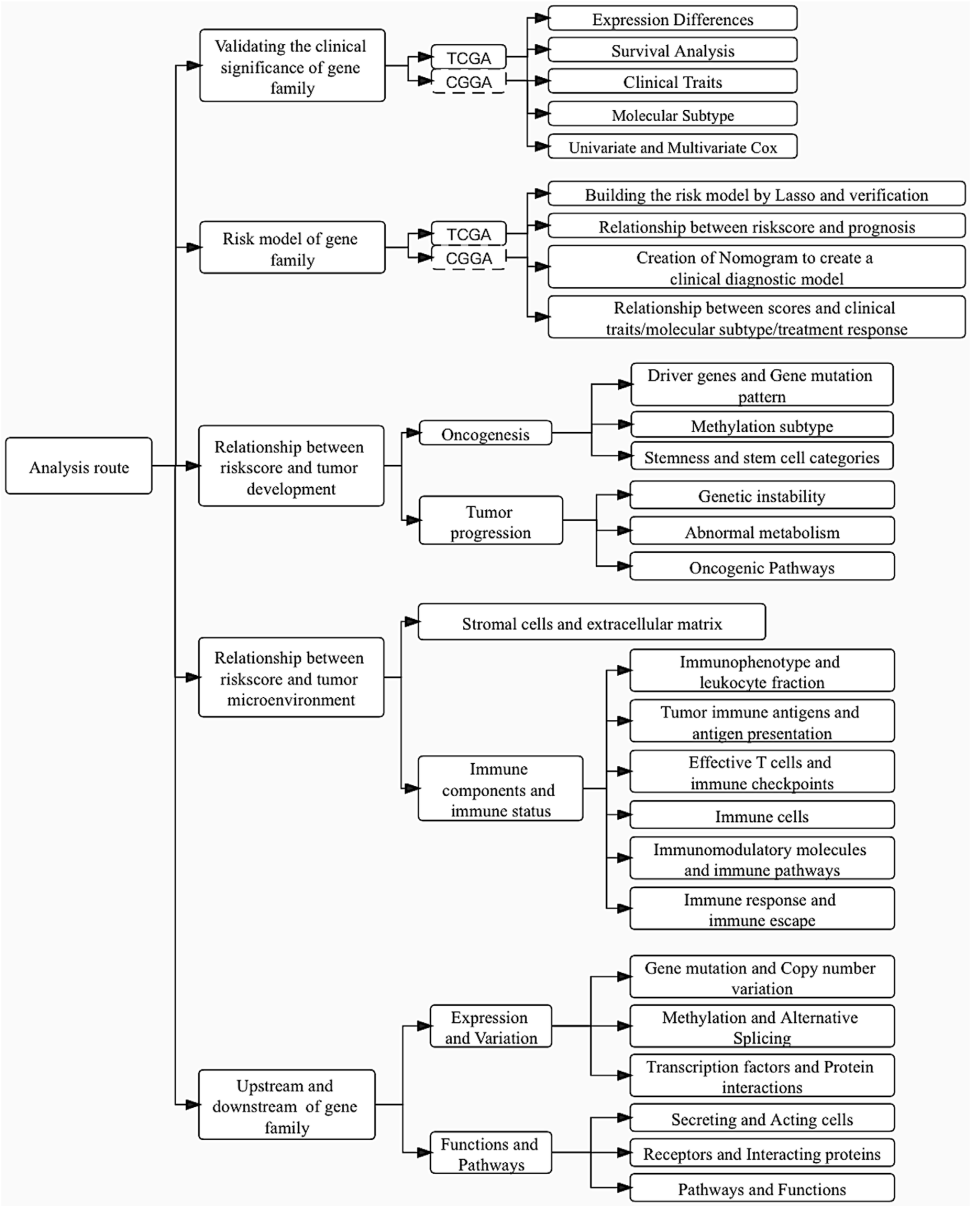
Article flowchart.

**FIGURE 2:**
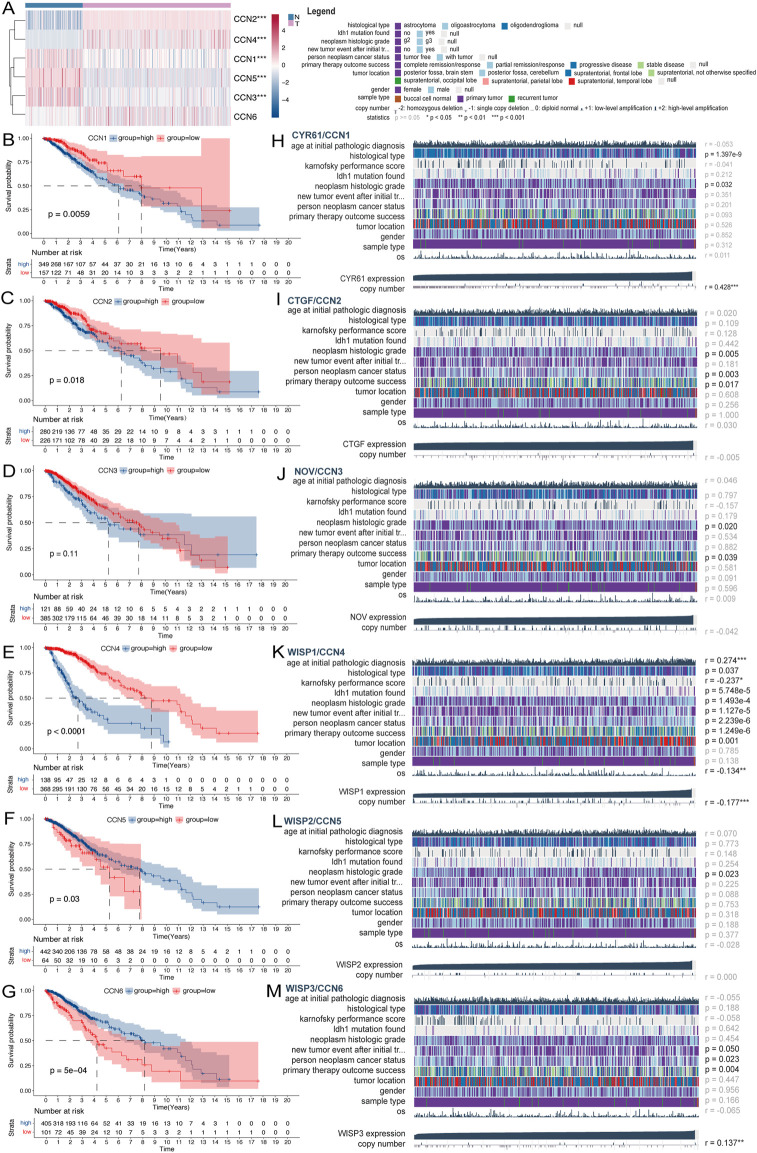
Expression and clinical value of the CCN family in LGG. **(A)** Heatmap of differential expression analysis of CCN family between normal cortex (*n* = 207) and TCGA-LGG cohort (*n* = 529). **(B–G)** Kaplan-Meier survival analysis was performed using the optimal cutoff value to distinguish the CCN genes between high and low subgroups in the TCGA-LGG cohort. **(H–M)** Correlation heatmap of CCN family expression *versus* clinical traits in the TCGA-LGG cohort using the MEXPRESS database. Each column in the graph represents a sample, while each row represents a variable. OS, overall survival. For all experiments, mean rank, *p < 0.05, **p < 0.01, ***p < 0.001.

A brief pipeline was represented as Figure 1. First, to identify the dysregulation of CCN in LGG, a differential analysis was conducted. Except CCN6, the expression of CCNs were significantly different between tumor and normal. CCN2 and CCN4 were highly expressed in tumor, and CCN1, 3, 5 were increased in the normal sample. These findings were validated by the CGGA-LGG cohort (Supplementary Figure S1A), indicating the dysregulation of CCNs in LGG. Then, the prognostic significance of CCN proteins was explored. Using the optimal cut-off value, significant survival differences was found in all genes except CCN3, where increased expression of CCN1, CCN2, and CCN4 predicted shorter survival, and increased expression of CCN5 and CCN6 predicted longer (Figures 2B–G, Supplementary Figure S1B–G). The relevance between CCN genes with clinical characteristics was also explored. Almost all CCN proteins were associated with pathological grade and first treatment response (Figure 2H–M). Notably, CCN4 was correlated with almost all clinical traits with prognostic impact. In addition, we found significant differences in the distribution of almost all CCN genes in supervised DNA methyaltion subtype, IDH/codel subtype and IDH/codel/TERTp subtype in TCGA database as well as in IDH/codel subtype, transcriptome subtype, primary and recurrent subtype of CGGA database, with similar distribution patterns for CCN1, CCN2, and CCN4. The expressions of CCN1, CCN2, and CCN4 were significantly increased in the IDH-wt group. In the IDH, 1p19q combined with TERT promoter subtype, we found that the expression of CCN4 was the highest in the TERTp-mut group, while CCN1 and CCN3 were mainly higher in the all negative group. This indicated that CCN4 was closely related to IDHwt and TERT promoter, which affected the prognosis of glioma. In supervised DNA methyaltion subtype, we found that CCN1, CCN2, and CCN4 were more highly expressed in classic-like and mesenchymal-like groups with poor prognosis than other groups. However, the expression of CCN1, CCN2, and CCN4 in codel group and G-CIMP-high group was significantly decreased. In addition, through CGGA data analysis, we found that CCN1, 2, 3, and 4 were all enhanced in relapsed glioma. CCN6 is the opposite. (Supplementary Figure S1H–M). In addition, CCN4 was an independent prognostic risk factor in univariate, multivariate cox analysis (Supplementary Figure S1N–R). In summary, these results suggested that CCN family genes were dysregulated in LGG and were associated with malignant clinical phenotypes and poor prognosis, especially CCN4.

### Construction and validation of CCNScore

**FIGURE 3:**
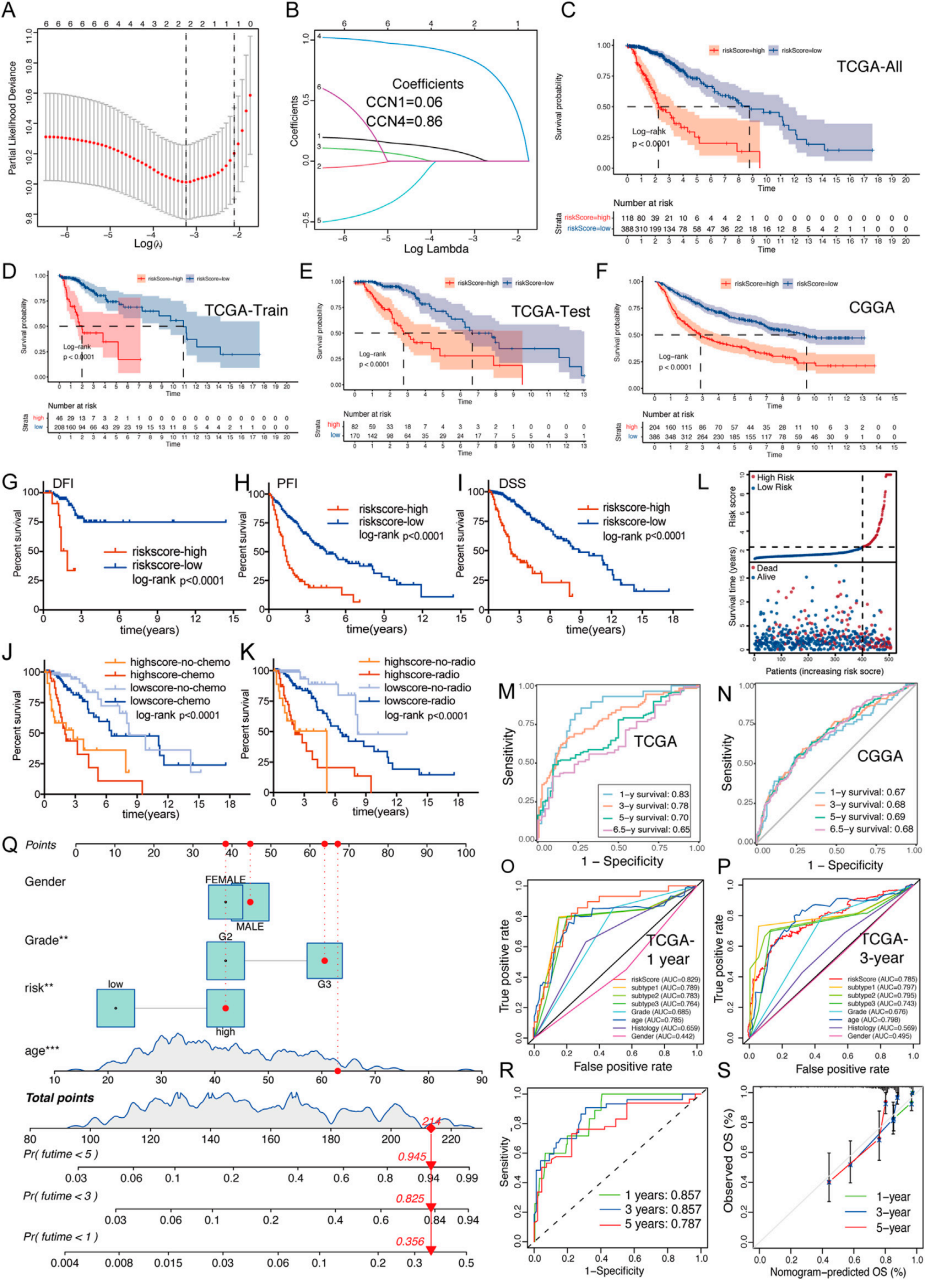
Creation and validation of CCNScore. **(A,B)** LASSO regression was performed, calculating the minimum criteria. **(C–F)** Overall survival analysis was performed of all TCGA-LGG set **(C)** and two robust clusters -TCGA train set **(D)** and TCGA test set **(E)**, as well as CGGA-LGG cohort **(F)** to validate CCNScore. **(G–I)** Survival analysis was performed using CCNscore, with DFI **(G)**, PFI **(H)** and DSS **(I)** as endpoint in the TCGA-LGG cohort. **(J,K)** Stratified survival analysis was performed according to radiotherapy **(J)**, chemotherapy **(K)** and CCNscore. **(L)** Risk curve of CCNscore was plotted using the TCGA-LGG cohort. **(M,N)** ROC curves indicated the risk prediction ability of CCNScore at 1, 3, 5, and 6.5 years in the TCGA-LGG cohort (M) and CGGA-LGG cohort (N). **(O,P)** ROC curves showed the accuracy of CCNscore vs. other clinical traits to predict prognosis at 1 and 3-year in the TCGA-LGG cohort. **(Q–S)** Nomogram based on CCNscore, age, gender and WHO grade **(Q)**. ROC indicates the ability of CCNscore to predict prognosis **(R)** and the corrected plot indicates the accuracy of predictive ability **(S)**. OS, Overall survival. DFI, disease free interval. PFI, progression free interval. DSS, disease-specific survival. **p < 0.01, ***p < 0.001.

LASSO regression analysis of CCNs was performed on the basis of the TCGA-LGG cohort to constructed a CCNScore. As a result, CCN1 and CCN4 were identified and their coefficients were calculated (CCN1 = 0.06, CCN4 = 0.86), characterizing CCNs in the most concise pattern (Figures 3A,B). Samples were classified into high-CCNScore subgroup and low-CCNScore subgroup based on the best cut-off score. Survival analysis showed that the high-CCNScore subgroup had worse overall survival (OS) (Figures 3C–F). Meanwhile, as LGG patients usually relapse, we also used disease-free interval (DFI), progression-free interval (PFI) and disease-specific survival (DSS) as clinical endpoints, and similar results were observed the high-CCNScore subgroup predicted a poor outcome (Figures 3G–I). Despite, our results showed the in the low-CCNScore subgroup, samples without radiotherapy had better outcome than those received radiotherapy (Figures 3J,K), suggesting that taking the CCNScore into account may further benefit patients. The risk-curve also indicated that significantly more patients died in the high-CCNScore subgroup (Figure 3L). Moreover, we did ROC analysis to evaluate the predictive accuracy of the CCNScore. In predicting 1-, 3-, 5-, and 6.5-year survival, CCNScore consistently showed a AUC >0.65 (Figure 3M,N), which performed better than other clinical traits (Figure 3O,P). Finally, a prognostic model was constructed based on the corresponding indictors, with the AUC values reached 0.857,0.857, and 0.787 in predicting 1-, 3-, and 5-year survival (Figure 3Q,S).

### Relationship between CCNScore and multiple clinical traits

**FIGURE 4:**
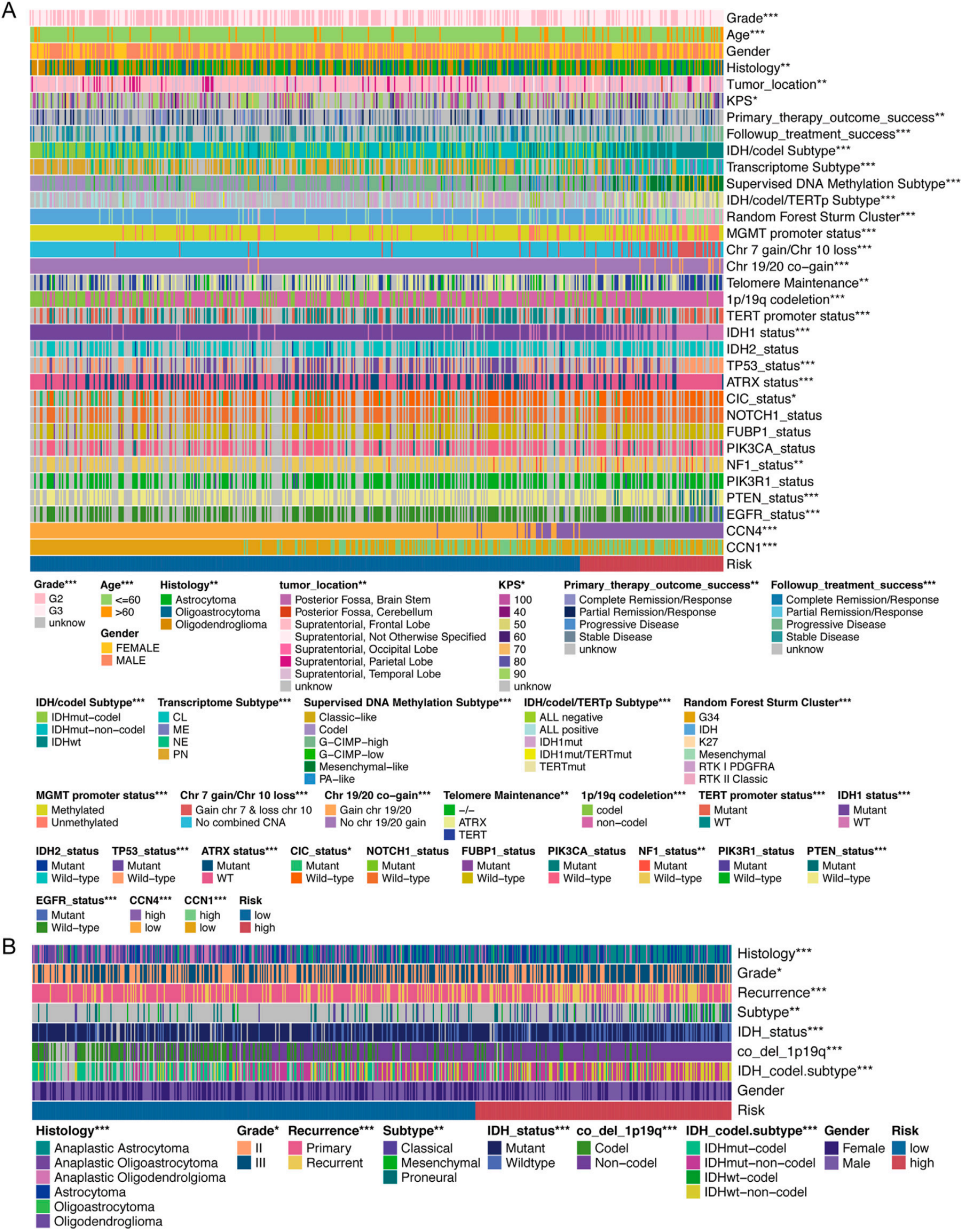
Relationship between CCNScore and clinical traits. **(A)** Correlation heatmap of CCNScore *versus* clinical traits in the TCGA-LGG cohort. **(B)** Correlation heatmap of CCNScore *versus* clinical traits in the CGGA-LGG cohort. *p < 0.05, **p < 0.01, ***p < 0.001.

We analyzed the relationship between the CCNScore and multiple clinical traits of LGG. The results showed that all except gender were correlated with the CCNScore, and the higher the CCNScore, the more concentrated the distribution of malignant phenotypes and the less sensitive to treatment, as validated by CGGA (Figures 4A,B). Moreover, we analyzed the relationship between CCNScore and LGG molecular phenotypes and found that the high-CCNScore group significant concentrated in IDHwt, Classic-like, mesenchymal-like, IDH1mut/TERTmut, MGMT promoter unmethylated, gain chr7and loss chr 10, gain chr 19/20, TERT telomere maintenance, TERT promoter mutant subgroup, as well as NF1mut, PTENmut and EGFRmut subgroup, showing a GBM-like molecular signature**.** Together, these results indicated that the high-CCNScore subgroup was associated with malignant phenotypes of LGG.

### Genomic and epigenetic phenotype of CCNScore-based subgroups

**FIGURE 5:**
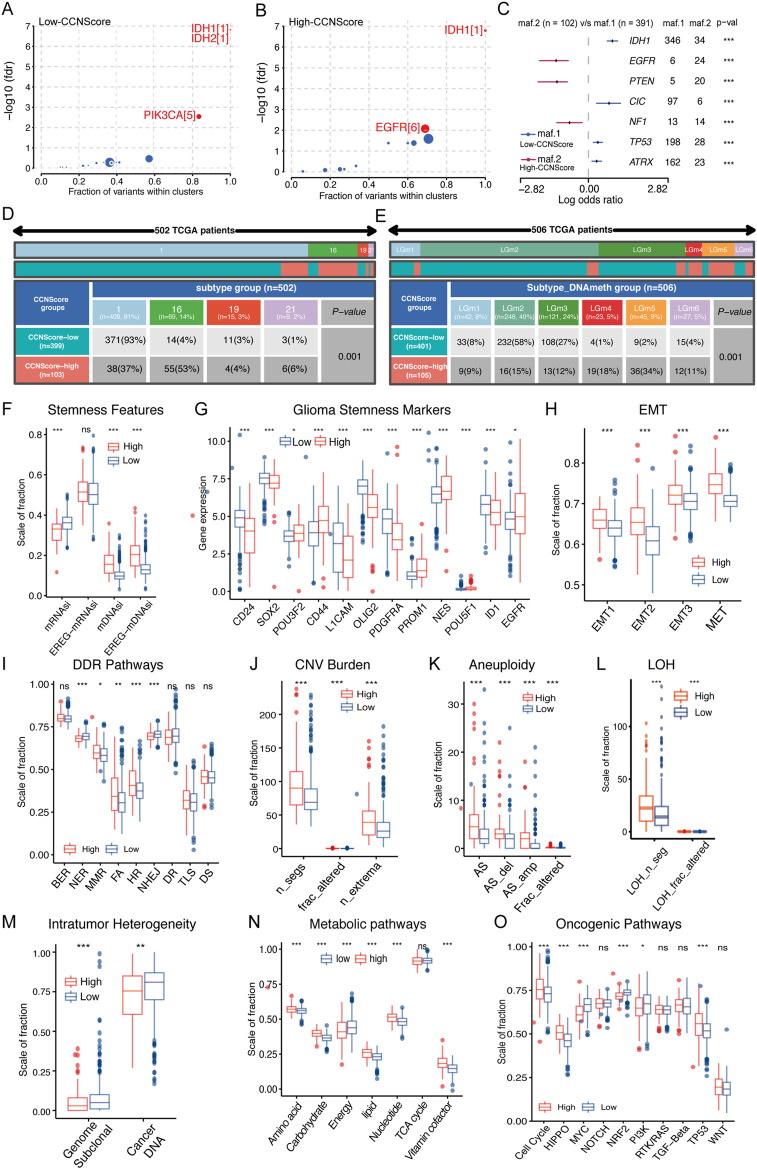
Relationship between CCNScore and the development of LGG. **(A,B)** Bubble plot shows the driver genes of Low-CCNScore **(A)** and High-CCNScore **(B)** groups. **(C)** Forest plot shows the differential mutation patterns in the Low-CCNScore and High-CCNScore groups. **(D,E)** Pan-cancer methylation subtypes **(D)** and Pan-glioma methylation subtypes **(E)** of Low-CCNScore and High-CCNScore groups. **(F–H)** Differences in stemness index **(F)**, markers of glioma stem cells **(G)**, and EMT *versus* MET phenotype **(H)** between Low-CCNScore and High-CCNScore groups. **(I–M)** Differences in DNA damage repair pathways **(I)**, copy number variation burden **(J)**, aneuploidy **(K)**, loss of heterozygosity **(L)** and intratumor heterogeneity **(M)** between Low-CCNScore and High-CCNScore groups. **(N)** Differences in the metabolic levels of seven major substances between the Low-CCNScore and High-CCNScore groups. **(O)** Differences in 10 oncogenic pathways between Low-CCNScore and High-CCNScore groups. BER, Base excision repair. NER, nucleotide excision repair. DR, direct damage reversal/repair. MMR, mismatch repair. HR, Homology-dependent recombination. NHEJ, non-homologous end joining. FA, Fanconi anemia. TLS, translation DNA synthesis. DS, damage sensor. For all experiments, mean rank, *p < 0.05, **p < 0.01, ***p < 0.001.

The genomic alterations underline CCNScore-based group was explored. Both groups had IDH mutations as putative tumor initiating factors, but the secondary driver diversed (Figures 5A,B). Interestingly, the gene mutation pattern of the two subgroups was different, with genes mutations of EGFR, PTEN, and NF1 enriched in the high-CCNScore group, and IDH1, CIC, TP53 and ATRX in the low-CCNScore group (Figure 5C). Similar results were obtained from waterfall plots (Supplementary Figure S2A,B). Thereafter, the association between CCNScore and significant mutant genes were identified, almost identical to the above results (Supplementary Figure S2C–J). In summary, the highCCNScore subgroup had a GBM-like mutation pattern.

Epigenomic dysregulation is also an important factor driving cancer development and progression. We found that the Low-CCNScore group mainly contained Type 1, while the High-CCNScore group mainly contained Type 16 of the pan-cancer methylation subtype (Figure 5D). Previous studies have shown that Type 1 was characterized by ATRX, TP53, and IDH1 mutations, and Type 16 contained mainly EGFR-driven LGG with PTEN mutation, in line with the above genomic results (Hoadley et al., 2018). Next, we found the Low-CCNScore subgroup was significantly enriched in the LGm2 and LGm3 groups, while the high-CCNScore subgroup had significantly higher numbers in the LGm4, LGm5, and LGm6 groups (Figure 5E). Previous studies have shown that the LGm1/LGm2/LGm3 groups were driven by IDH mutation and showed genome-wide hyper-methylation, while the LGm4/LGm5/LGm6 groups were IDH wild type (Ceccarelli et al., 2016). In summary, our results showed that the low-CCNScore subgroup was characterized by high methylation level, and the high-CCNScore group was the opposite.

### CCNScore is associated with stemness, genetic instability and metabolism dysregulation of lower-grade glioma

Stemness is crucial to tumor progression. In terms of stemness indices, CCNScore positively correlated with mDNAsi and EREG-mDNAsi, and negatively correlated with mRNAsi, possibly because of LGG-specific IDH1 mutations leading to hypermethylation (Figure 5F, Supplementary Figure S2K). In terms of GSCs markers, the high-CCNScore subgroup had increased expression of PROM1, CD44, EGFR, and POU3F2, and decreased expression of OLIG2, SOX2, CD24, ID1, PDGFRA, and L1CAM (Figure 5G, Supplementary Figure S2L). Different markers were associated with different GSCs types, with CD24 being highest in neural progenitor cells (NPC)-like cells, CD133 in oligodendrocyte-progenitor cells (OPC)-like cells, EGFR in astrocytes (AC)-like cells and CD44 in mesenchymal state (MES)-like cells, and NES showed a clear preference for AC-like cells (Suva and Tirosh, 2020). Therefore, our results suggested that OPC-like, AC-like and MES-like cells were mainly present in the high-CCNScore subgroup, whereas NPC-like malignant cells were predominant in the low-CCNScore group. Finally, for the EMT and MET processes linked to stemness, our results showed that both EMT and MET processes were enhanced in the high-CCNScore group, especially MET (Figure 5H), indicating that these cells can switch between a quiescent, dormant state and a migratory, mesenchymal-like state.

Loss of DNA damage repair (DDR) function leading to genetic instability and a risk of carcinogenesis. Our results showed that mismatch repair (MMR), Fanconi anemia (FA), Homology-dependent recombination (HR) processes and diminished nucleotide excision repair (NER) and non-homologous end joining (NHEJ) processes were enhanced in the high-CCNScore subgroup, in line with the results of the correlation heatmap (Figure 5I, Supplementary Figure S2N). Loss of specific DDR pathways in cancer often leads to stable DNA damage phenotypes. We found that intra-tumor heterogeneity (ITH) was lower in the high-CCNScore subgroup, while loss of heterozygosity (LOH), CNV and aneuploidy were all significantly higher (Figure 5J–M, Supplementary Figure S2N). It was shown that specific CNVs and LOH were associated with poor prognosis in LGG (Knijnenburg et al., 2018). Therefore, our results suggested that the high-CCNScore was associated with genetic instability and led to poor prognosis of LGG patients through a specific genetic damage pattern.

We analyzed seven important metabolic processes and found that energy metabolism was down-regulated in High-CCNScore group, and the rest were almost up-regulated (Figure 5N, Supplementary Figure S2O). Previous studies showed that poor prognosis of LGG was significantly associated with upregulation of carbohydrate, nucleotide, vitamin and cofactor metabolism, which fits with increased demand for glucose uptake and nucleotide synthesis. While downregulation of energy metabolism was associated with poor prognosis of LGG (Peng et al., 2018). Evidently, CCNScore was involved in a variety of LGG metabolic disorders that affect the prognosis of LGG. In addition, we found that High-CCNScore was positively associated with HIPPO, cell cycle, WNT, and TP53 pathways and negative correlated with MYC, NRF2 pathways by analyzing 10 typical tumor pathways (Figure 5O, Supplementary Figure S2P). In summary, we found that CCNScore, representing the CCN family, played a very important role in the development of LGG.

### CCNScore was associated with stromal and immune components in tumor microenvironment

**FIGURE 6:**
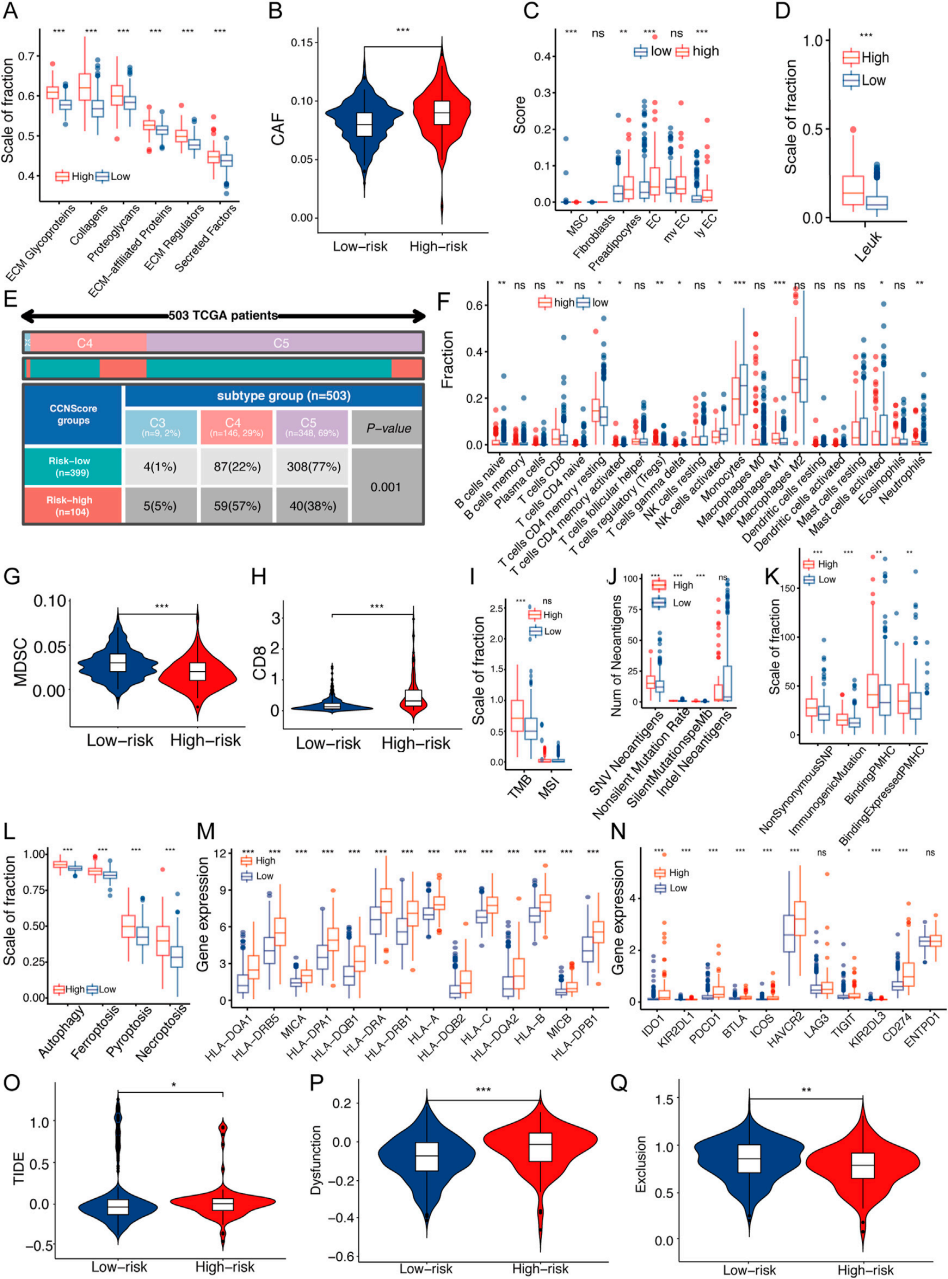
Relationship between CCNScore and TME of LGG. **(A)** Differences in extracellular matrix components of TME in Low-CCNScore and High-CCNScore groups. **(B,C)** Differences in CAF **(B)** and normal stromal cells **(C)** of TME in Low-CCNScore and High-CCNScore groups. **(D)** Differences in leukocyte fraction of TME between Low-CCNScore and High-CCNScore groups. **(E)** The proportion of Low-CCNScore and High-CCNScore among pan-cancer immune subtypes. **(F)** Differences in tumor-infiltrated immune cells of TME between Low-CCNScore and High-CCNScore groups. **(G,H)** Differences in MDSC **(G)** and CD8 **(H)** in TME between Low-CCNScore and High-CCNScore groups. **(I–K)** Differences in tumor mutational burden and microsatellite instability **(I)**, SNV neoantigen count **(J)** and antigenic peptide-major histocompatibility complex **(K)** of Low-CCNScore and High-CCNScore groups. **(L)** Differences in four immunogenic deaths in TME of Low-CCNScore and High-CCNScore groups. **(M)** Differences in the expression of major histocompatibility complexes between Low-CCNScore and High-CCNScore groups. **(N)** Differences in the expression of immune checkpoints in TME between Low-CCNScore and High-CCNScore groups. **(O–Q)** Differences in immune escape **(O)**, immune dysfunction **(P)**, and immune exclusion **(Q)** between Low-CCNScore and High-CCNScore groups. CAF, cancer-associated fibroblasts. MDSC, myeloid-derived suppressor cell. For all experiments, mean rank, *p < 0.05, **p < 0.01, ***p < 0.001.

ECM stiffness is closely related to the tumor malignancy, and as a regulatory protein of ECM, the effects of CCNScore on ECM was evaluated. Our results showed that all ECM-associated components were significantly elevated in the high-CCNScore group (Figure 6A). ECM is mainly derived from stromal cells, so we estimated the content of cancer-associated stromal cells (CAFs) and several stromal cells in TME. The results showed that CAFs were significantly increased in the high-CCN4 group (Figure 6B, Supplementary Figure S3A), and in addition, we found that vascular-associated stromal cells were also significantly elevated in the high-CCNScore subgroup, especially endothelial cells (Figure 6C). In summary, these results suggested that CCN genes were associated with CAF and endothelial cells, which induce ECM remodeling.

To understand the effect of the CCN family on the immune microenvironment of LGG, we analyzed the association between the CCNScore and immune characteristics. As a result, the leukocyte fraction (LF) was significantly higher in the high-CCNScore group than the Low-CCNScore group, demonstrating that high-CCNScore had increased immune infiltration (Figure 6D). The proportion of pan-cancer immunophenotypes in the high-CCNScore group *versus* the low-CCNScore group were explored, and we found that the Low-CCNScore group clustered mainly in the C5 phenotype, while the High-CCNScore group aggregated significantly in the C4 phenotype (Figure 6E). Previous studies have shown that C4 was lymphocyte-depleted, exhibiting prominent macrophages with suppressed TH1 cells, and C5 was immune-quiet that had the best prognosis among these subtypes, showing the lowest lymphocytes and the highest macrophages with M2 predominance (Thorsson et al., 2018). These results indicated that the immune activation was stronger in the high-CCNScore group, which was associated with poor prognosis of LGG.

Secondly, we used multiple analysis software to investigate the relationship between CCNScore and immune infiltration. Overall, the High-CCNScore group was characterized by a significant decrease in monocytes and myeloid-derived suppressor cell (MDSC), and an increase in macrophages, CD8^+^T cells, and myeloid dendritic cells (Figures 6F–H, Supplementary Figure S3A,B).

Thirdly, neoantigens is a powerful activator of anti-tumor immunity. Therefore, we analyzed tumor mutational burden (TMB), single nucleotide variants (SNV) neoantigens, including insertional deletion mutations (indel), silent and non-silent mutations, major histocompatibility complex-related genes (MHC), MHC-binding SNV-derived peptides (pMHC) and immunogenic death including autophagy, necroptosis, ferroptosis and pyroptosis. As a result, the high-CCNScore had strong antigenicity and enhanced antigen presentation (Figure 6I–M).

In addition, for immune molecules, we found that most of the immuno-stimulatory genes were significantly increased in the high-CCNScore group. Interestingly, the immuno-suppressive genes, immune checkpoints and various immune-related pathways were also almost all higher in the high-CCNScore group (Figure 6N, Supplementary Figure S3C–E). It has been suggested that enhanced immunosuppression may be a compensatory response to enhanced immune activation, so tumors with high inflammatory response are enriched in pro- and anti-inflammatory factors (Doucette et al., 2013). Taken together, these results suggested that there was an increased inflammation in the high-CCNScore group.

Finally, since tumor cells can evade recognition and immune attack through immune editing, we analyzed the overall process of immune escape, immune dysfunction and immune exclusion by TIDE database. We found that the low-CCNScore group had higher immune response, while the high-CCNScore group had higher immune dysfunction and immune escape (Figure 6O,Q). We found that the high-CCNScore group scored higher in T cell dysfunction and TIDE, indicating an impaired immune activity and high immune escape.

### A multi-omics overview and functions of the CCN family in lower-grade glioma

**FIGURE 7:**
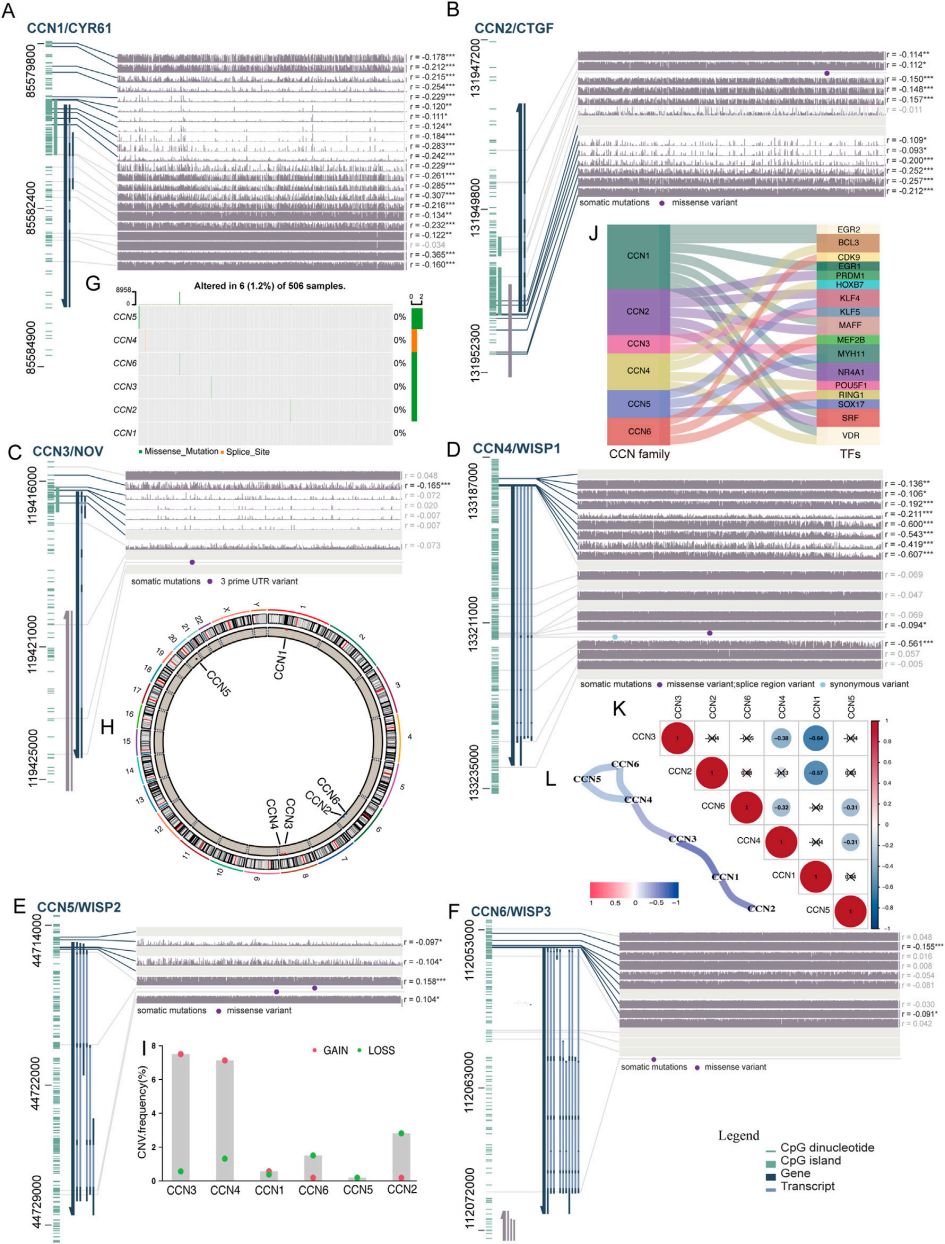
A landscape of multi-omics changes of the CCN family in LGG. **(A–F)** Overview of methylation, transcripts and mutations of the CCN family in the TCGA-LGG cohort. Each row shows the DNA methylation data for a single probe on the Infinium microarray, the darkened line indicates promoter methylation. The gene together with its transcripts, as well as any CpG islands and all the individual CpG dinucleotides are shown on the left, and the r value indicates the correlation between gene methylation and expression. r: Pearson correlation coefficient. **(G)** Waterfall plot showing CCN family mutations in 506 LGG patients from the TCGA-LGG cohort. The upper bar graph shows the tumor mutation burden for each patient. The number on the right indicates the mutation frequency of the CCNs gene, and the bar on the right shows the patient number for each mutation type. **(H)** Circle diagram showing the copy number variation and location of CCNs genes on 23 chromosomes using the TCGA-LGG cohort. Red dots on the outer ring indicate amplification and blue dots on the inner ring indicate deletion. **(I)** CNV frequency plot of the CCN family in the TCGA-LGG cohort. **(J)** Sankey diagrams represent tumor-associated promoters of CCNs genes. **(K)** Correlation analysis of protein interactions of the CCN family. **(L)** Line diagram showing protein interactions between the CCN family. *p < 0.05, **p < 0.01, ***p < 0.001.

To exhibit the CCN family in multi-omics scale, we first investigated gene mutations. The CCN family genes were rarely mutated in LGG (1.2% altered) (Figure 7G). We also explored the copy number variants, and found that CCN1, CCN3, and CCN4 mainly showed amplification, and CCN2 and CCN6 mainly showed deletion (Figures 7H,I). At the epigenetic level, the methylation at several locations of CCN1, CCN2, and CCN4 were negatively correlated with expression (Figures 7A–F). At the transcriptional level, we found significant differences in CCNs associated cancer transcription factors in LGG *versus* normal brain tissue, with most being decreased, but HOXB7, the promoter of CCN4, was significantly increased 4.4-fold more than normal brain tissue (Figure 7J, Supplementary Table S2), demonstrating that promoter changes were an important part involved in the dysregulation of CCN genes expression. At the post-transcriptional level, we showed the number of CCN family transcripts (Figures 7A–F). Finally, at the protein level, we found CCN proteins showed a general antagonistic manner (Figures 7K,L).

In addition, to find the function and pathway of CCN proteins, we analyzed their upstream and downstream (Supplementary Figure S4A). Single-cell RNA-seq analysis showed that CCN4 was mainly associated with AC-like, MES-like cells and endothelial cell. Next, we showed proteins that interact with CCN genes (Supplementary Figure S4B). Multiple integrins that act as CCN proteins receptors were upregulated in LGG (Supplementary Table S3) and positively correlated with CCN proteins (Supplementary Figure S4C). Moreover, the CCN proteins were associated with hypoxia, ROS production, mTOR, extracellular matrix organization, EMT, angiogenesis, autophagy and apoptosis by analyzing hallmarks of tumor, GO and KEGG (Supplementary Figure S5A–C). The above results demonstrated that CCN proteins lead to multiple malignant phenotypes closely associated with tumor progression through binding to different integrins in LGG. Taken together, these results suggested that the expression pattern of the CCN family was mainly regulated by CNV, methylation levels and promoter changes, and regulated LGG progression through multiple malignant pathways.

## Discussion

First, we verified the differential expression and the prognostic significance of CCN proteins in LGG. Second, The CCNScore based on the CCN family was first established in this paper and was crucial for prognosis prediction, subtype evaluation, and treatment selection. Next, we analyzed and characterized the genomic, epigenetic profiles and tumor microenvironment characteristics, *etc.* Of patients in High-CCNScore and Low-CCNScore groups, and confirmed that the High-CCNScore group had a GBM-like genetic pattern, associated with high stemness, high genomic instability, high inflammation, high ECM phenotype, metabolic disorders and poor prognosis. Finally, we outlined the multi-omics variation as well as pathways and functions of the CCN family. Our comprehensive analysis of the CCN family demonstrated the important role of the stromal regulatory proteins in LGG and provided a new perspective for improving the treatment of LGG.

CCN proteins are a subset of ECM and play an important regulatory role. They are highly conserved and their dysregulation occurs mostly when inflammation or tissue damage becomes chronic, which is also a risk factor for cancer development (Perbal, 2004). It has been shown that CCN1, CCN2, and CCN4 all contribute to glioma progression (Yin et al., 2010; Gaudreau et al., 2019; Uneda et al., 2021), in agreement with our findings. A riskscore model we constructed based on the CCN family for the first time in LGG has important clinical applications. First, based on the CCNScore we created a prognostic score system that can predict patient survival over time by combining age, gender and pathological grade. Second, the classification of glioma has evolved from simple histomorphology to a combination of histomorphology and molecular biology, and because of the heterogeneity of cells within glioma, combined with the fact that the morphology of different types of gliomas can overlap with each other (Sottoriva et al., 2013; Wang et al., 2017), so a more accurate classification is needed to reflect the biological characteristics of gliomas. As shown in our results, CCNScores can better distinguish patients with long-term survival of LGG from those with high-grade glioma features that are likely to deteriorate rapidly than other indicators. Finally, CCNScore is important to guide the choice of therapy for different patients, for patients with low scores, better benefits will be obtained without opting for radiotherapy.

Cancer is thought to result from uncontrolled cellular behavior caused by genetic mutations, so identifying the mutations that drive cancer development and progression is the basis for elucidating the biological characteristics of cancer (Martinez-Jimenez et al., 2020). It has been shown that LGG patients with IDH mutations accompanying 1p19q deletions have features of oligodendrogliomas, 62% have CIC mutations, and 96% will carry TERT promoter mutations. Patients with IDH-mut without 1p19q deletion almost always have TP53 mutations (94%) with ATRX (86%) inactivation and are rarely accompanied by TERT mutations (Bettegowda et al., 2011; Liu et al., 2012; Yip et al., 2012; Cancer Genome Atlas Research et al., 2015). While IDH wild-type LGG closely resembles glioblastoma (GBM) in both molecular signatures and clinical behavior, often including PTEN, EGFR and NF1 mutations (Verhaak et al., 2010; Brennan et al., 2013). Consistent with our results, at the genomic level, the Low-CCNScore group can be described as a merger of two LGGs with IDH-mut, while the High-CCNScore group can be described by a GBM-like mutation pattern, and also has a poor GBM-like prognosis. In addition, previous studies have shown that IDH mutations are accompanied by hyper-methylation, while patients with IDH-mut without 1p19q deletion partially exhibit hypo-methylation and are associated with a poorer prognosis (Mur et al., 2013). Moreover, IDH-wt gliomas also correspond to three methylation clusters, including classical-like, mesenchymal-like, and pilocytic astrocytoma-like (PA-like) subtypes. Among them, the PA-like subtype, which has almost no mutations in EGFR, CDKN2A/B and PTEN (Ceccarelli et al., 2016). We concluded that the High-CCNScore group mainly included the classic-like and mesenchyma-like IDH-wt LGG with low methylation levels, while the Low-CCNScore group mainly included codel and the G-CIMP-high subtype, with a better prognosis.

GSCs are an important factor contributing to glioma progression and recurrence, and we first investigated the stemness index, which represents the degree of stemness. Previous studies have shown that mDNAsi is positively correlated with advanced pathological grade of gliomas and that mDNAsi is mainly driven by hypo-methylation levels and mesenchymal subtypes. In addition, mDNAsi is elevated in mutations in NF1 and EGFR, but negatively correlated with IDH1, TP53, CIC, and ATRX mutations (Malta et al., 2018). Consistent with our findings, we suggest that the Low-CCNScore group resembles a non-stem phenotype with hyper-methylated characteristics. While the High-CCNScore group resembles G-CIMP-low tumors and exhibits a greater proliferative capacity and has stem cell-like genomic signatures (Ceccarelli et al., 2016). In addition, Weiwei Tao et al. (2020) showed that CCN4 has the role of maintaining tumor stem cells to further confirm our view. Secondly, we further investigated the types of CCNs-associated GSCs, and since proliferation in IDH-mut gliomas is mainly restricted to NPC-like cells, this means it may limit the speed of tumor growth. AC-like cells appear to have the potential to reduce tumorigenesis, whereas MES-like, NPC-like and OPC-like cells are associated with glioma recurrence and aggressiveness (Suva and Tirosh, 2020). Therefore, we suggest that the Low-CCNScore group has GSC restriction, while the High-CCNScore group has GSC multiplicity, and that the induction of AC-like state may have therapeutic implications for glioma (Suva et al., 2014). Furthermore, the EMT process is inextricably linked to tumor stemness, and our results showed that CCN proteins consistently correlated closely with EMT and MET. Therefore, we speculate that CCN proteins may enhance LGG stemness by enhancing the EMT and MET pathway.

Studies have shown that the composition of TME in brain tumors can be influenced by the molecular characteristics (Wang et al., 2017). First, we found that the Low-CCNScore group was mainly concentrated in the C5 subtype. It has been shown that 80% of IDH mutations are enriched in C5 (Thorsson et al., 2018), while IDH mutations can lead to a decrease in tumor-associated immune cells and better prognosis by reducing leukocyte chemotaxis (Venteicher et al., 2017), which is consistent with the Low-CCNScore group. Second, both chromosome 1p deletion (including TNFRS9 and VTCN1) and 19q deletion (including TGFB1) were associated with lower LF, which is consistent with the role of TGF-β in immune cell recruitment (Batlle and Massague, 2019), all of which indicate a molecular profile consistency of TME characteristics in the Low-CCNScore group. Our results showed a significant increase in macrophages in the High-CCNScore group, and previous studies have shown that 30% of immune cells in brain tumors are macrophages (Graeber et al., 2002), and the number of TAMs is inversely correlated with tumor survival and positively correlated with tumor grade (Hambardzumyan et al., 2016). Moreover, deficiency of NF-1 in IDH-wt gliomas leads to increased macrophage recruitment and is seen most frequently in the mesenchymal subtype (Wang et al., 2017). TAMs include tissue-resident microglia as well as bone marrow-derived macrophages (BMDMs) (Bowman et al., 2016). And BMDMs play a major role in supporting the growth of tumor-initiating cells (Chen et al., 2017). Our results showed that the increase in macrophages was accompanied by a significant decrease in monocytes in the High-CCNScore group, so we suggested that the CCNs-associated TAMs were mainly composed of BMDMs. In addition to macrophages, we also found an increase in myeloid-derived dendritic cells and CD8 cells in the High-CCNScore group. The increase in CD8T cells was not related to any molecular subtype but to the hyper-mutated phenotype (Wang et al., 2017), as these tumors may produce more neoantigens that can be recognized by T cells, in agreement with our results.

In addition to the composition of immune cells, the structure of the brain extracellular matrix (ECM) seems to help distinguish the characteristics of LGG and HGG. The structure and composition of the brain ECM is unique compared to other organs and tissues, containing mainly heparin sulfate proteoglycan (HSPG) and hyaluronic acid (HA) (Perus and Walsh, 2019). Studies have shown that there is a significant increase in HSPG production in glioma and the dense ECM leads to hypoxia and tumor aggressiveness (Wade et al., 2013; Lemjabbar-Alaoui et al., 2015). A recent study showed that ECM stiffness correlates with increased glioma grade, while IDH-mut glioma exhibit reduced aggressiveness associated with reduced ECM stiffness and mechanical signaling, and in addition, IDH-mut glioma can mediate decreased tenascin-C and HA levels through down-regulation of HIF-1α (Miroshnikova et al., 2016), suggesting that differences in ECM composition in glioma are partially regulated by IDH mutational status. Since the ECM scaffolds the tissue and regulates cell structure and inflammation, ECM differences between IDH-mut and IDH-wt may in part be the basis for the differences in the landscape between TME of LGG and HGG, and consequently influence the evolution of the disease. Furthermore, recent studies have shown that as ECM proteins, CCNs can be tethered to other ECM proteins, including decorin, fibronectin, vitronectin and perlecan, and can act as a local scaffold that coordinates the interaction of specific bioactive molecules, ECM proteins and target cells leading to extracellular matrix remodeling (Jun and Lau, 2011). Consistent with our study that high CCNScore is associated with significantly elevated stromal proteins, and the high ECM phenotype contributes to a variety of biological processes and glioma progression.

Metabolic reprogramming is considered one of the hallmarks of cancer (Hanahan and Weinberg, 2011; Kaymak et al., 2021). Glioma is a metabolically affected disease due to IDH mutation (Dang et al., 2009), and LGG shows the broadest survival correlation with metabolic disorders compared to other tumors (Vander Heiden and DeBerardinis, 2017). Furthermore, metabolic activities are intrinsically linked to cancer marker pathways, such as carbohydrate metabolism positively correlated with angiogenesis, EMT and inflammation upregulation (Peng et al., 2018). Previous studies have shown a general increase in mTORC1 signaling for all metabolic subtypes except energy metabolism (Hay, 2016), and our results suggest a significant positive correlation between CCNs and mTORC1 signaling. Surprisingly, recent studies have shown that in a mouse glioma model, mTOR activity is restricted to the few cell layers closest to the perfused vessels, and that cancer cells within this perivascular layer are characterized by intense anabolic metabolism, and perivascular cancer cells exhibited enhanced tumorigenicity, migratory and invasiveness, as well as unexpectedly specific chemo- and radio-therapy resistance, all in a mTOR-dependent manner. This mTOR-mediated compartmentalized metabolism directly influences the acquisition of multiple aggressive tumor hallmarks (Kumar et al., 2019). The relevance of stromal cell proteins to metabolism has been rarely investigated before, and in this paper, we demonstrated for the first time the relevance and importance of CCNs to tumor metabolic disorders, which helps further expand the understanding of the biological function of CCNs and provides another promising therapeutic target for LGG.

There are still some limitations in this study. First, we used the functional class scoring (FCS) approach to quantify phenotypes and pathways, but FCS analyzes each pathway independently, and since the same gene may be involved in multiple pathways, it may lead to significant enrichment of individual pathways due to overlapping genes. In addition, treating each gene as an individual ignores the biological properties of genes and the complex interactions between genes. Secondly, it is the protein, that is, the basic unit that performs the function, and our studies are at the transcriptome level, so the impact of post-transcriptional modifications, and post-translational modifications need to be further explored. Finally, our study mainly demonstrates correlations, which need to be combined with basic experiments to verify upstream and downstream and direct interaction relationships.

## Conclusion

In this paper, we validated the clinical value of the CCN family in LGG and constructed the first CCN family-based riskscore system to guide clinical application. In addition, we explored the biological functions of CCNs in LGG, and we found that CCNs were also closely associated with gene mutations, high-inflammation, high-ECM, high-stemness, metabolic abnormalities and immune escape in LGG, in addition to malignant phenotypes such as tumor proliferation and invasion, and has important and specific research value in LGG.

## Data Availability

The original contributions presented in the study are included in the article/Supplementary Material, further inquiries can be directed to the corresponding authors.

## Supplementary Material

SUPPLEMENTARY FIGURE S1
Validation of the clinical significance of the CCN family. **(A)** Heatmap of differential expression analysis of CCN family between normal cortex (*n*=207) and CGGA-LGG cohort (*n*=590). **(B–G)** Kaplan-Meier survival analysis was performed using the optimal cutoff value to distinguish the CCN genes between high and low subgroups in the CGGA-LGG cohort. **(H–J)** Box plots showed the expression of CCN family genes in glioma DNA Methylation subtypes **(H)**, IDH/codel subtypes **(I)** and IDH/codel/TERTp subtypes **(J)** in the TCGA-LGG cohort. **(K–M)** Box plots showed the expression of CCN family genes in IDH/codel subtype **(K)**, transcriptome subtype **(L)**, and primary or recurrence subtype **(M)** in the CGGA-LGG cohort. **(N–O)** Forest plots showed univariate cox analysis **(N)** and multivariate cox analysis **(O)** of the CCN family in the CGGA-LGG cohort. **(P–Q)** Forest plots showed univariate cox analysis **(P)** and multivariate cox analysis **(Q)** of the CCN family in the TCGA-LGG cohort. **(R)** ROC curves indicated the prognostic risk prediction of CCN4 at 1, 3, and 5 years in the TCGA-LGG cohort. **p* < 0.05, ***p* < 0.01, and ****p* < 0.001.

SUPPLEMENTARY FIGURE S2
Correlation between CCN family and the development of LGG. **(A,B)** Somatic mutation waterfall plots were created based on high-CCNScore group **(A)** and low-CCNScore group **(B)**. **(C–J)** Column charts show the proportion of patients with mutant type and wild type of important genes in the Low-CCNScore and High-CCNScore group. **(K–P)** The correlation heatmap showed the correlation between CCN family genes and CCNscore with stemness signature **(K)**, GSCs markers **(L)**, DNA damage repair pathway **(M)**, genomic change profile **(N)**, substance metabolism **(O)** and oncogene pathways **(P)**. **p* < 0.05, ***p* < 0.01, and ****p* < 0.001.

SUPPLEMENTARY FIGURE S3
Role of the CCN family in the TME of LGG. **(A)** Correlation of CCNScore expression with tumor-infiltrated immune cells and stromal cells was analyzed in each software using multiple databases. **(B)** Correlation heatmap showed the association of CCN family and CCNscore with the immune cells calculated by CIBERSORT. **(C–E)** Differences in expression of immune-stimulatory genes **(C)** and immune-suppressive genes **(D)** and tumor microenvironment-related pathways **(E)** between Low-CCNScore and High-CCNScore groups. **p* < 0.05, ***p* < 0.01, and ****p* < 0.001.

SUPPLEMENTARY FIGURE S4
Receptors and Interacting Proteins of CCN family. **(A)** Single cell sequencing analysis of correlation between CCN4 and immune stromal cells and malignant cells. **(B)** Protein-protein interaction networks of CCN family genes were constructed using the STRING database. **(C)** Correlation of CCN family and CCNscore with integrins. **p* < 0.05, ***p* < 0.01, and ****p* < 0.001.

SUPPLEMENTARY FIGURE S5
Functions of CCN family. **(A)** Correlation of CCN family and CCNscore with tumor hallmarks. **(B,C)** KEGG **(B)** and GO **(C)** analysis of the CCNScore high and low subgroups indicated the main functions and pathways of CCN family. The barplot was order by q value. **p* < 0.05, ***p* < 0.01, and ****p* < 0.001.
