# Supplementary figures and images for: Stromal protein CCN family contributes to the poor prognosis in lower-grade gioma by modulating immunity, matrix, stemness, and metabolism
Source: Front Mol Biosci. 2022 Dec 16;9:1027236. doi: 10.3389/fmolb.2022.1027236 (PMC9800986; doi:10.3389/fmolb.2022.1027236)

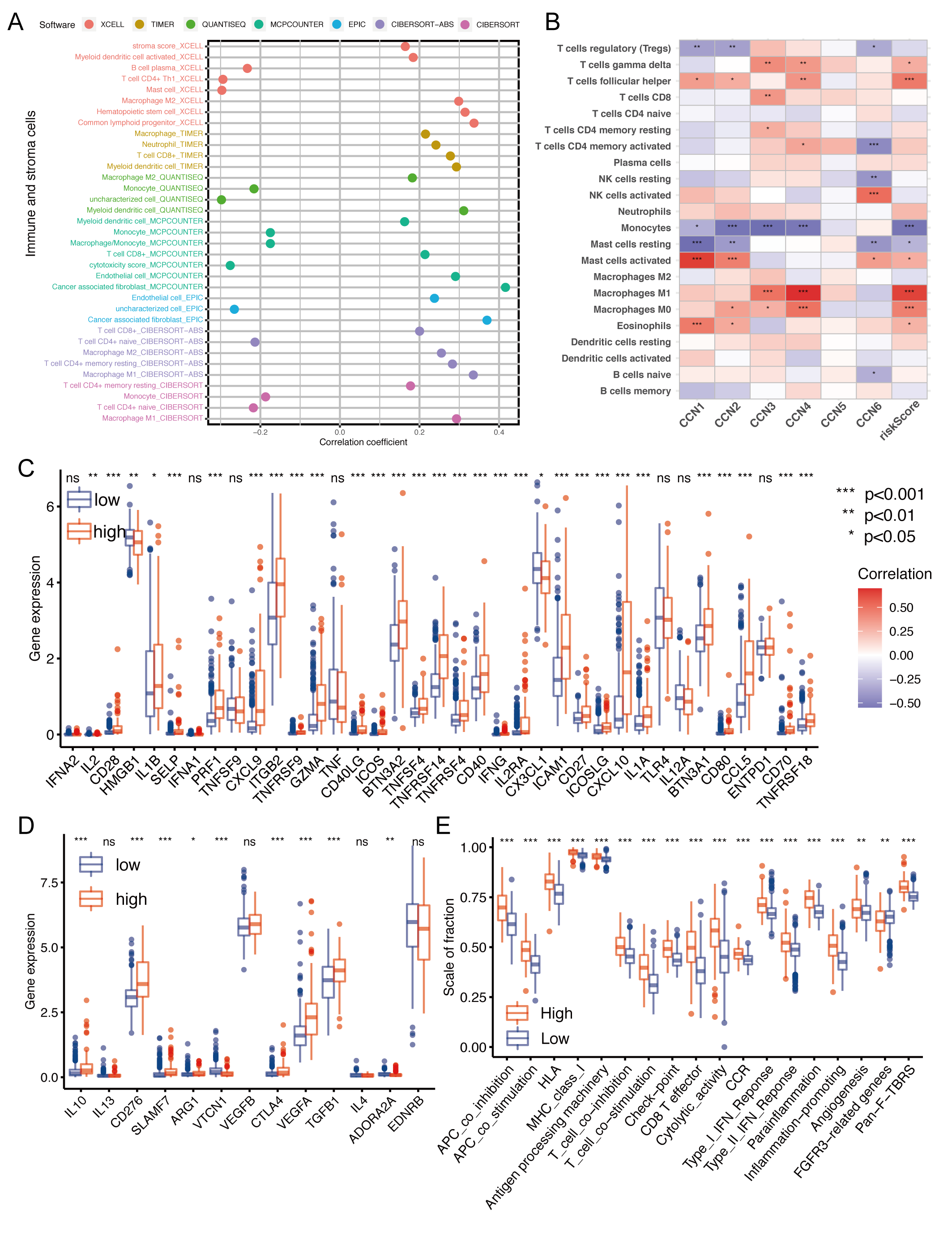

Supplement: Supplementary file 3 [file Image3.TIF]

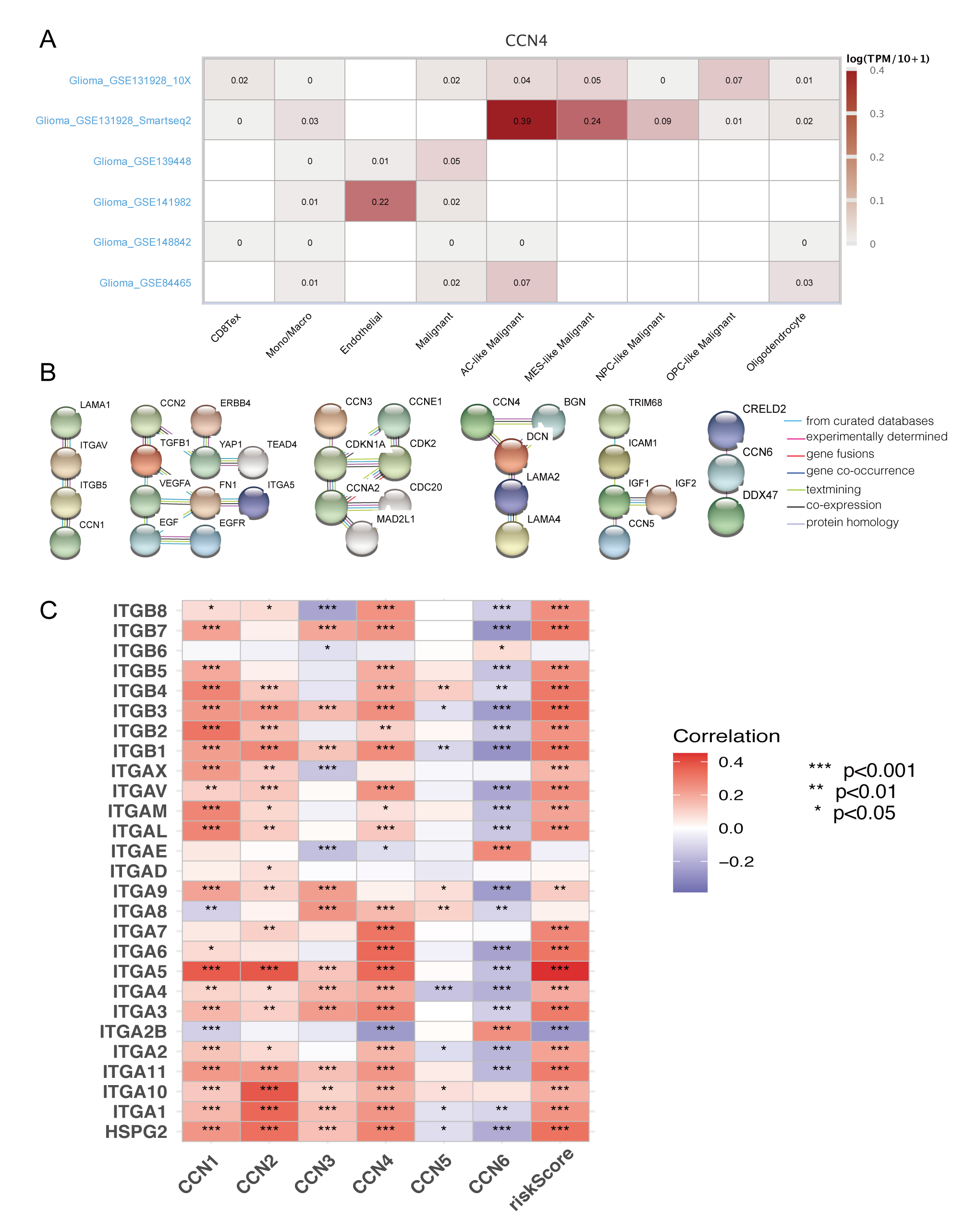

Supplement: Supplementary file 4 [file Image4.TIF]

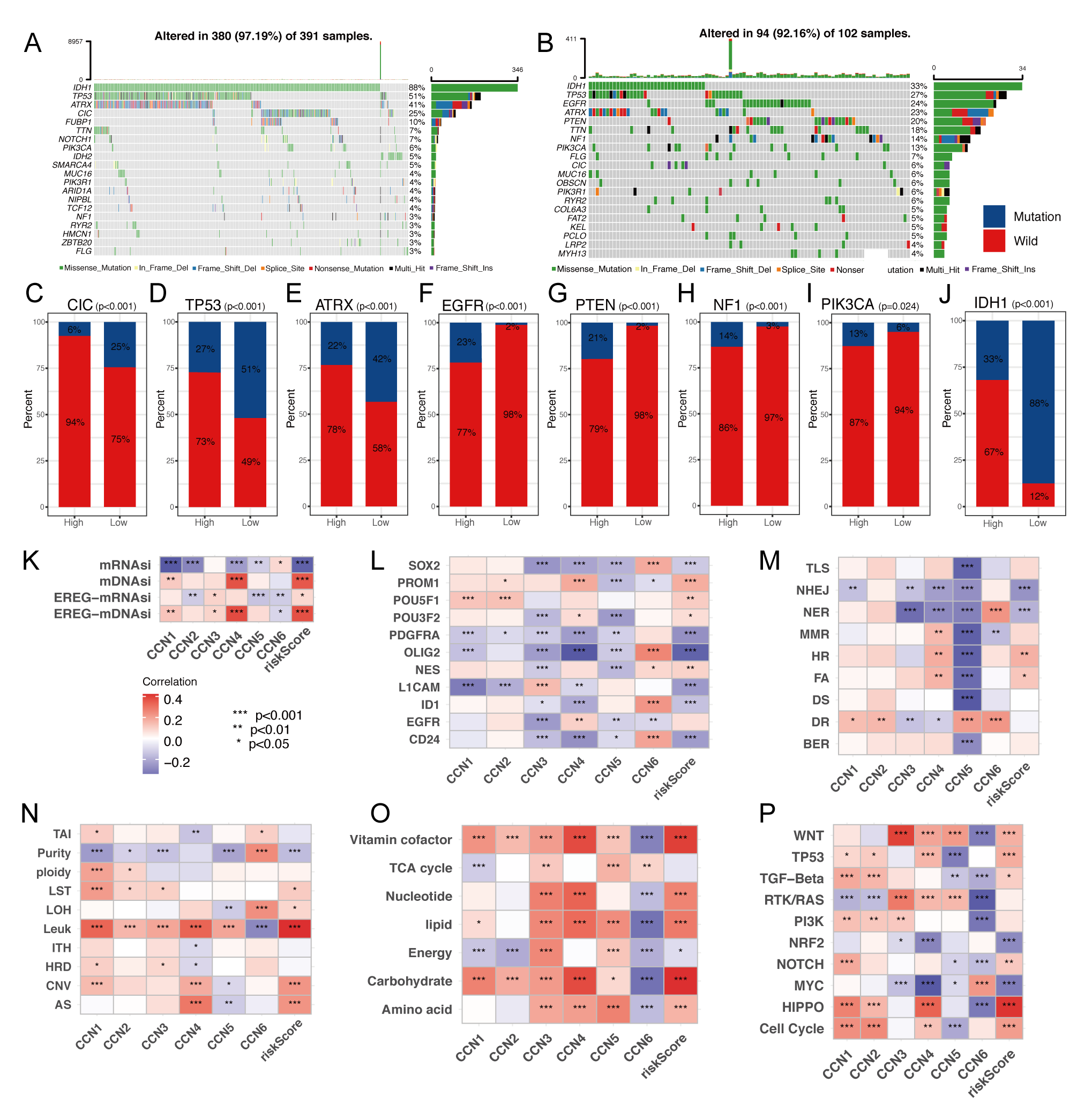

Supplement: Supplementary file 5 [file Image2.TIF]

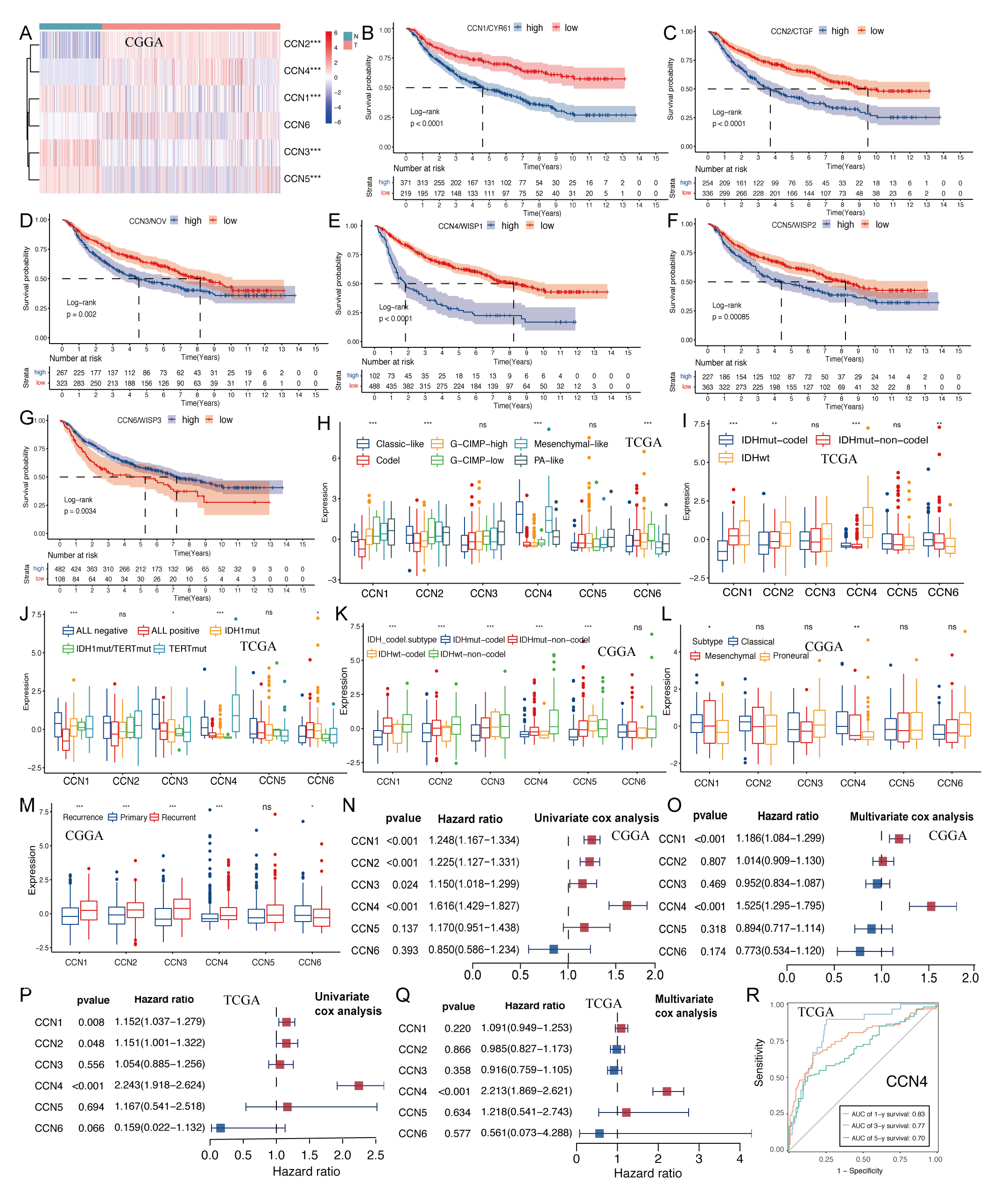

Supplement: Supplementary file 6 [file Image1.TIF]

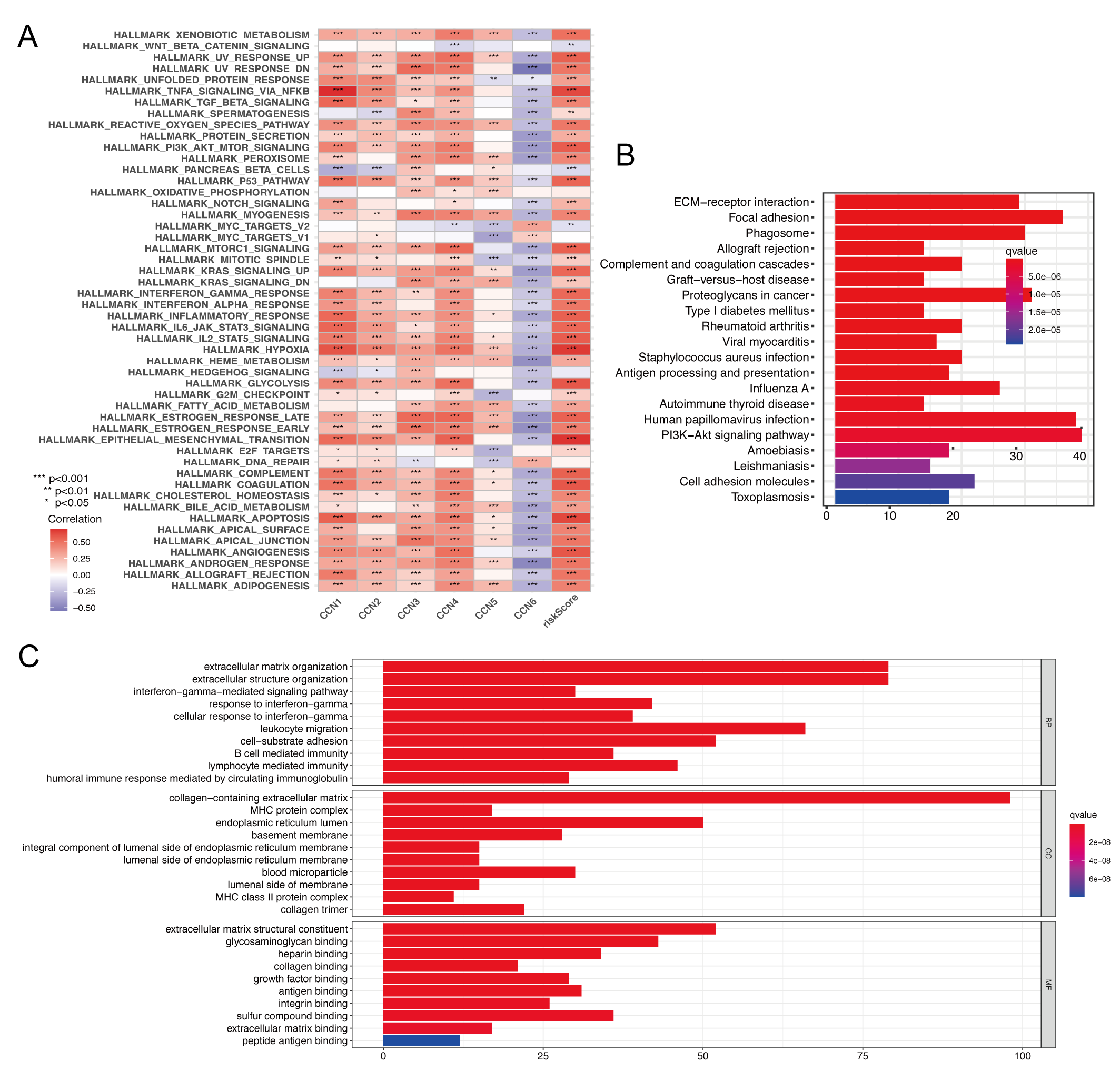

Supplement: Supplementary file 8 [file Image5.TIF]
